# Advance of Stem Cell Treatment for Traumatic Brain Injury

**DOI:** 10.3389/fncel.2019.00301

**Published:** 2019-08-13

**Authors:** Yunxiang Zhou, Anwen Shao, Weilin Xu, Haijian Wu, Yongchuan Deng

**Affiliations:** ^1^Department of Surgical Oncology, The Second Affiliated Hospital, School of Medicine, Zhejiang University, Hangzhou, China; ^2^Department of Neurosurgery, The Second Affiliated Hospital, School of Medicine, Zhejiang University, Hangzhou, China

**Keywords:** traumatic brain injury, stem cell, mechanism, treatment, review

## Abstract

Traumatic brain injury (TBI) is an important cause of human mortality and morbidity, which can induce serious neurological damage. At present, clinical treatments for neurological dysfunction after TBI include hyperbaric oxygen, brain stimulation and behavioral therapy, but the therapeutic effect is not satisfactory. Recent studies have found that exogenous stem cells can migrate to damaged brain tissue, then participate in the repair of damaged brain tissue by further differentiation to replace damaged cells, while releasing anti-inflammatory factors and growth factors, thereby significantly improving neurological function. This article will mainly review the effects, deficiencies and related mechanisms of different types of stem cells in TBI.

## Introduction

Traumatic brain injury (TBI) is a common and frequently occurring disease. According to the World Health Organization, TBI will become the main cause of human mortality and morbidity after 2020, which brings a heavy economic burden to patients and families (Maas et al., 2017). TBI is a disease which causes the destruction of normal brain function, and leads to serious physical, cognitive and emotional disorders. The pathophysiology of TBI mainly includes the break of the blood brain barrier (BBB), extensive neuroinflammation, diffuse axonal injury, and neurodegenerative lesions (Xiong et al., 2008). The pathological changes of brain injury are mainly the loss of normal tissue structure, destruction of neuronal cells and internal environment disturbance, among which neuronal cells injury is the key point. There is no effective drug treatment so far. At present, the main treatment includ hyperbaric oxygen, non-invasive brain stimulation, task-oriented functional electrical stimulation and behavioral therapy (Dang et al., 2017).

In recent years, study (Cox, 2018) have found that a variety of stem cells can treat neurological impairment after TBI, including mesenchymal stem cells (MSCs), neural stem cells (NSCs), multipotent adult progenitor cells (MAPCs), and endothelial progenitor cells (EPCs) (Table 1). In this article, we review the literature on the role, progress, major deficiencies and related mechanisms of different types of stem cell therapy in TBI.

**TABLE 1:** The TBI models and the roles of stem cells involved in the literature.

**References**	**TBI Model injury was induced by**	**Injury location**	**Cell Culture and Animals**	**General results**	**Potential mechanism**	**Target protein/pathway**
Zhang et al., 2013	A weight-drop hitting device with a 4.5-mm-diameter cylinder bar weighing 40 g from a height of 20 cm	The right cortex midway between the lambda and the bregma	MSC and Sprague-Dawley rats	The neurological function of TBI animals in the MSCs treatment group was significantly improved from 3 to 28 days	Anti-inflammatory and immunomodulatory	The secretion of TSG-6 by MSC suppresses NF-κB signaling pathway
Guo S. et al., 2017	A 20 mg steel rod with a flat end and a diameter of 2 mm drop on a piston resting on the dura from a height of 50 cm	Sagittal suture between bregma and lambda	MSC and C57BL/6 male mice	The recovery of neurological function, learning and memory ability were improved, and neuronal apoptosis was reduced in the MSCs treatment group	Promoting angiogenesis and improving neurological function	Diminish activation of caspase-3, upregulate expression of vascular endothelial growth factor and angiogenin-1
Shi et al., 2018	A pneumatic piston impactor tip (3 mm diameter) as penetration depth was 1.0 mm and the velocity was 4.5 m/s	The right skull	MSCs transduced with SOD2 adenovirus. And male adult Balb/c mice (6–8 weeks)	The neurological function was improved in the MSCs with SOD2 over-expressed treatment group	SOD2 can reduce the neuroinflammatory response, maintain the integrity of BBB and attenuate neuro-inflammation of the ipsilateral cortexin in TBI mice	NA
Zhang et al., 2015	A pneumatic piston containing a 6-mm-diameter tip at a rate of 4 m/s and 2.5 mm of compression	Left cortex	Exosomes derived from MSCs and adult male Wistar rats	MSC-generated exosomes effectively improve functional recovery	Promoting endogenous angiogenesis and neurogenesis, reducing inflammation and brain inflammation in rats after TBI	Targeting multiple targets
Zhang et al., 2017	The same as Zhang et al. (2015)		The same as Zhang et al. (2015)	Exosomes cultured in three-dimensional collagen have better effects in improving neurological recovery and spatial learning ability after TBI	Exo-3D may be attributed to further enhanced neurogenesis and reduced activation of microglia/astrocytes in the DG compared to the Exo-2D group	NA
Haus et al., 2016	A pneumatic controlled cortical impact device with a 5 mm flat metal impactor tip at a rate of 4.5 m/s, 2.5 mm of compression and a dwell time of 500 ms	Left cortex, centered over hippocampus	NSCs and adult male athymic nude rats (NCI RNU -/- homozygous, 10–11 weeks old)	The recovery of cognitive function after brain injury was observed for a long time in the NSCs treatment group	Improving hippocampal neuron survival	NA
Philips et al., 2001	A rapid pulse of saline that struck the exposed dura through the sealed fitting with a lateral FPI of moderate severity (2.3–2.4 atm)	The left parietal bone centered between the coronal, sagittal, and lambdoid sutures	NSCs transfected with a virus carrying a nerve growth factor gene and adult male Wistar rats	Significantly increased pyramidal cell survival in the hippocampus, and enhanced the ability of cognitive, learning, and motor function	By the transplanted cells themselves and the secreted transgenic nerve growth factor	NA
Bedi et al., 2013a	A 6-mm-diameter flat impactor tip, a single impact of 2.7 mm depth of deformation with an impact velocity of 5.6 m/s and a dwell time of 150 ms (moderate-severe injury)	The midpoint between bregma and lambda, 3 mm lateral to the midline, overlying the temporoparietal cortex	MAPCs and male rats weighing 250–300 g	MAPCs can improve their spatial learning, information retention, memory retrieval and dyskinesia after 120 days of brain injury, and can maintain the integrity of the blood-brain barrier in the acute phase of traumatic brain injury	Anti-inflammatory	NA
Walker et al., 2010	A single impact of 3.1 mm depth of deformation with an impact velocity of 5.8 m/s and a dwell time of 150 ms (moderate–severe injury)	The midpoint between bregma and lambda, 3 mm lateral to the midline, overlying the temporoparietal cortex	MAPCs and male Sprague-Dawley rats rats weighing 225–250 g	The intravenous injection of MAPC preserves the integrity of the blood brain barrier	Modulating the systemic immunologic and inflammatory response via interactions with other organ systems such as splenocytes.	NA
Walker et al., 2012	A single impact of 1.0-mm depth of deformation with an impact velocity of 5.0 m/s and a dwell time of 150 ms (moderate-severe injury)	The location is unclear	MAPCs and C57B6 mice	Significant increases in the splenocyte, plasma T regulatory cell populations and the brain M2/M1 macrophage ratio were observed with MAPC therapy	Direct contact between the MAPC and splenocytes	NA
Kobayashi et al., 2012	Spinal cord injury marmoset model		iPSCs and adult female common marmosets	Functional recovery was promoted in iPSCs treatment group	Promoting axonal regeneration and preventing brain tissue damage	NA
Lyu et al., 2017	An impact from a 20 cm high position along the guide bar by a 50 g hammer, which resulted in a predominantly focal injury of the right cerebral cortex	l 2.5 mm away from the sagittal suture and 1.5 mm away from the arcuate suture	A2B5+ iPSCs and female Sprague-Dawley rats, weighing 200 to 240 g	Neurological function was improved in A2B5+ iPSCs treatment group	Modulating the expression of lncRNA and mRNA	NA
Wei et al., 2016	An electric impact device with the impact (velocity = 3 m/s, depth = 2.0 mm, contact time = 150 ms) led to evident damage in the cortical regions, specifically the sensorimotor cortex	Midway between lambda and bregma, 2.0 mm to the right of the central suture	iPSC-derived neural progenitor cells and male P14 Wistar rats	Performance in social interaction, social novelty, and social transmission of food preference tests were improved in hypoxic preconditioning- iPSC-neural progenitor cells	(1) increasing HIF-1a; (2) upregulating downstream regenerative factors such as BDNF, GDNF, vascular endothelial growth factor, and/or erythropoietin; and (3) increasing expression of social behavior genes such as oxytocin and the oxytocin receptor	NA
Dunkerson et al., 2014	Controlled cortical impact injury	Medial frontal cortex	Combining enriched environment and iPSCs. adult male rats	Motor performance were improved, and full cognitive restoration was seen	NA	NA
Huang X. T. et al., 2013	A fluid percussion instrument with the impacted pressure set to 1.5e1.8 atm	2.0 mm posterior from bregma and 1.5 mm lateral to the sagittal suture	ECFCs and adult female BALB/C nude mice (8 weeks of age)	Formation of new vessels, neurological functions and BBB integrity were improved	Repairing disrupted BBB and enhancing angiogenesis in the host brain	
Ran et al., 2015	A PinPoint precision brain injury impactor, the dura mater was impacted at 3 m/s to induce craniocerebral injury	Midpoint between bregma	EPCs and adult specific-pathogen-free male Wistar rats	Neurovascular repair was promoted in Notch-signaling-pathway-activated endothelial progenitor cells	Enhancing the migration, invasiveness and angiogenic ability of endothelial progenitor cells.	The Notch signaling pathway
Guo X. B. et al., 2017	The fluid percussion device	4.0 mm posterior from bregma and 3.0 mm lateral to the sagittal suture	EPCs and adult male Wistar rats (weight: 300–350 g)	Neurological function after TBI was improved	Promoting hippocampal neurogenesis and angiogenesis	NA
Xue et al., 2010	A footplate of a 4.5 mm diameter tip at force of 20 g × 30 cm (force is expressed as weight × distance dropped) and 2 mm of compression.	The left hemisphere, the center of the bone-hole was positioned 2 mm anterior and 2.5 mm lateral to the bregma	EPCs and 2-month-old adult male Wistar rats weighing 220–280 g	Functional recovery was improved, deficiency volume of brain was reduced	Participating in capillary formation, reducing astrocyte proliferation and inflammation	NA
Chen et al., 2013	A 20 g poise from 50 cm-high place, contacting with cylindrical hammer (4 mm diameter and 3 mm high), stucking the pachymeninx, a moderate cortical impact	Between the bregma and lambdoid suture	EPCs and healthy Sprague-Dawley rats	The brain injury was diminish	Restoring cerebral blood perfusion and increasing the cerebral microvasculature	NA
Park et al., 2014	A moderate extradural FPI with the weighted pendulum set to an angle of 251 resulting in a fluid pressure pulse of 2.5 atmospheres with 15-ms duration	The midline suture, midway between the bregma and lambda	EPCs and adult male Sprague-Dawley rats (350 to 400 g)	Less degeneration of postischemic axons was seen in EPCs treatment group	Mediating local angiogenesis in the brain, maintaining the integrity of white matter	NA

*MSCs = mesenchymal stem cells; NSCs = neural stem cells; MAPCs = multipotent adult progenitor cells; EPCs = endothelial progenitor cells; ECFCs = endothelial colony-forming cells; TBI = traumatic brain injury; TSG-6 = TNF-α stimulated gene/protein 6; NF-κB = nuclear factor-κB; SOD2 = superoxide dismutase 2; iPSCs = induced pluripotent stem cells; NA = not available; GDNF = glial cell line-derived neurotrophic factor; BDNF = brain-derived neurotrophic factor; BBB = blood brain barrier; FPI = fluid percussion injury.*

## Therapeutic Effect of Different Types of Stem Cells

### Mesenchymal Stem Cells

**FIGURE 1:**
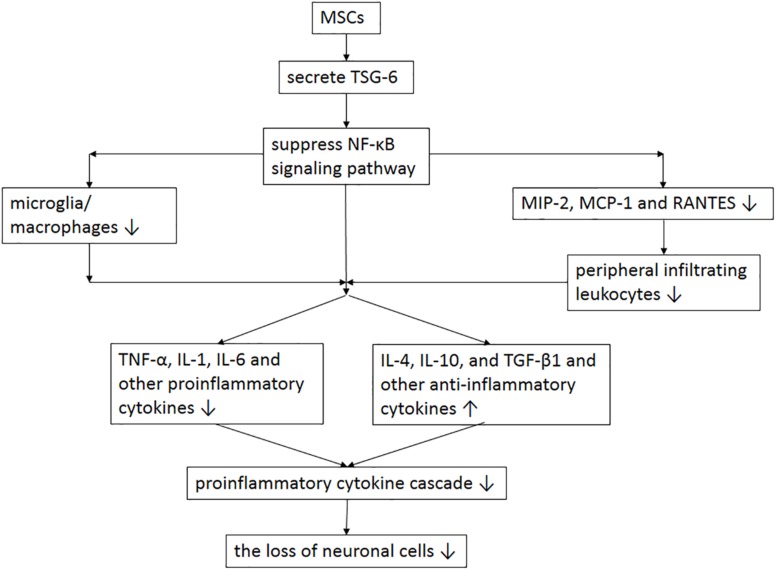
The potential mechanism of MSCs involved in anti-inflammatory and immunomodulatory after TBI. MSC transplantation enhances the expression of TSG-6 and then suppresses the activation of NF-κB signaling pathway, resulting in the reduction of microglia, macrophages, peripheral infiltrating leukocytes and proinflammatory cytokines and the increase of anti-inflammatory cytokines, which thereby alleviate the initiation of proinflammatory cytokine cascade and the loss of neuronal cells. MSCs = mesenchymal stem cells; TBI = traumatic brain injury; TNF = tumor necrosis factor; TSG-6 = TNF-α stimulated gene/protein 6; NF-κB = nuclear factor-κB; IL = interleukin; MCP = macrophage chemotactic protein; MIP = macrophage inflammatory protein; TGF = transforming growth factor; RANTES = regulated on activation in normal T-cell expressed and secreted.

Mesenchymal stem cells are heterogeneous multipotent adult cells that can be isolated from bone marrow, and perivascular tissues (da Silva Meirelles et al., 2006; Wong et al., 2015), with the ability of directional differentiation into mesenchymal and non-mesenchymal tissues, including nerve cells (Sanchez-Ramos et al., 2000). MSCs play an important role in tissue regeneration, which can promote the regeneration of damaged tissues by inhibiting inflammation, secreting trophic factors, and recruiting local progenitor cells to replace lost cells. Study (Adibi et al., 2016) has shown that MSCs can down-regulate the expression of inflammatory proteins and accelerate the repair of intracranial aneurysms. Moreover, study (Wang et al., 2013) has found that in addition to its secretory ability, MSCs can selectively migrate to the injured brain tissue of the TBI rat, and then differentiate into neurons and astrocytes to repair damaged brain tissue, thereby improving the motion function after TBI. Zhang et al. (2013) used the TBI rat model to study the anti-inflammatory and immunomodulatory properties of MSCs. Compared with the control group, the neurological function of TBI animals in the MSCs treatment group was significantly improved from 3 to 28 days, with the brain water content decreased significantly. In addition, they found that MSCs treatment could reduce the number of microglia, macrophages, neutrophils, CD3+ lymphocytes, apoptotic cells and pro-inflammatory cytokines in the injured cortex, thereby inhibiting the inflammatory response after TBI (Figure 1) (Zhang et al., 2013). Similarly, Guo S. et al. (2017) studied the treatment effect by injecting bone marrow-derived MSCs into TBI mice. Compared with the control group, MSCs treatment could promote the recovery of neurological function in TBI mice, improve learning and memory ability, and reduce neuronal apoptosis. The mechanism may be that MSCs promote the expression of VEGF and Ang-1, and microangiogenesis. Despite simply transfecting MSCs into the body, injecting MSCs with proliferation and anti-oxidative effects enhanced through overexpression of specific gene *in vitro* is also an efficient treatment for TBI. Furthermore, superoxide dismutase 2 (SOD2) plays a critical role against oxidative stress, Shi et al. (2018) found that SOD2 could reduce the neuroinflammatory response and maintain the integrity of BBB in TBI mice; and by intravenously transfecting MSCs, in which the SOD2 gene is overexpressed, into TBI mice, the neurological function could be ultimately improved. Although basic research (Djouad et al., 2003) has confirmed that MSCs can improve the neurological function and prognosis of TBI, MSCs using in clinical practice is still difficult, as treatment with MSCs has the potential to promote brain tumor growth. To date, there have been two clinical studies on the efficacy and safety of MSCs in the treatment of TBI. In one of the clinical studies (Zhang et al., 2008), seven patients were transplanted with MSCs during craniocerebral surgery. The surgeons injected MSCs directly to the injured brain tissue, and venously injected 10^8^ to 10^10^ MSCs into TBI patients; neurological function improved significantly at 6 months follow-up, and no evidence of toxicity was found. However, the sample size of this study was too small and there was no control group, and the intervals between MCSs transplantation and injury of direct infusion and intravenous infusion were different, while the interval has an important correlation with homing effect. Moreover, the numbers of MSCs injected into each patient were very different, without regularity or explanation. No quantitative or qualitative analysis of the aggregation effect of MSCs on the injury site were recorded. The second one was Phase I clinical study (Wang et al., 2017) including 10 patients with severe brain trauma. MSCs were injected intravenously or intrathecally. The improvement of neurological function was mainly determined by NIHSS scale, GCS score and GOS score. After the cells were transplanted, the serum nerve growth factor and brain-derived neurotrophic factor were significantly increased, and during the 6 months follow-up, the patients’ neurological function was improved, and no death or adverse events occurred. However, the limitation of, this clinical study is similar to the clinical study mentioned above as the sample is small and there is no control group, and is not as comprehensive and rigorous in the evaluation of the adverse effect. Although MSCs have achieved good therapeutic effects in both basic and clinical research, their ability to survive for long periods on the TBI model and the possible presence of immunological rejection are important issues that warrant further investigation (Gold et al., 2013). Currently, cell replacement, long-term transplantation-mediated nutritional support or immune regulation are the main mechanisms of the effect of MSCs. However, low cell viability, immune rejection, and inability to quantify cell viability may affect the accurate assessment of the ability of MSCs for long-term repair and recovery of neurological function (Haus et al., 2016).

*In vitro* exosomes are small vesicles containing various RNAs (mRNAs, miRNAs, etc.) and proteins with a diameter of 30–100 nm, which can be released from multiple cells under normal or pathological conditions (Barteneva et al., 2013). Exosomes have important clinical value, as their surface markers and molecules can be used as potential diagnostic markers for some diseases (Escudero et al., 2016), which not only have low immunity and long half-life in the peripheral circulation, but also have the ability to cross the BBB (Escudero et al., 2016). Compared with other types of cells, MSCs can produce a large number of exosomes, and the extracted exosomes are not significantly different from other cell-derived exosomes in morphological characteristics, isolation and storage methods (Yeo et al., 2013). In addition to transplantation of MSCs into TBI animal models by different routes, Zhang et al. (2015) extracted exosomes from MSCs and then injected exosomal proteins through the tail vein to the TBI animal. Results showed that neurological function in the TBI group could be significantly improved, and the mechanism might be related to the promoting endogenous angiogenesis and neurogenesis, as well as reducing inflammatory response post-TBI. Then, by comparing the difference in the efficacy of exosome cultured in two-dimensional and three-dimensional collagen scaffolds, they found that exosomes cultured in three-dimensional collagen had better effects in improving neurological recovery and spatial learning ability after TBI (Zhang et al., 2017). Similarly, a recent study (Yeo et al., 2013) found that treated with exosomes from MSCs could alleviate the damage of cognitive function after TBI. Xin et al. (2013) found that exosomes could significantly promote the recovery of neurological function in rat stroke models. Further studies showed that compared with the control group, exosomes can enhance axonal reconstruction, neurogenesis, and angiogenesis. Compared with transplantation of exogenous stem cells, MSCs-derived exosomal transplantation has several advantages in repairing damaged brain tissue, for example:no ethical problems, less invasiveness, lower or no immunogenicity, and low or no tumorigenicity (Xiong et al., 2017). So far, there is no clinical study on the treatment of TBI using MSCs-derived exosomes. The underlying mechanism of TBI treatment of exosomes is still not fully understood, so the mechanism of exosome repairing damaged brain tissue after TBI need further study. It is a necessary step in its application to the clinic.

### Neural Stem Cells

Neural stem cells are self-renewing stem cells that can be further differentiated into neurons, glial cells and oligodendrocytes. Long-term survival of human cells transplanted into TBI animal models is difficult to achieve due to host immune rejection, and Haus et al. (2016) produced a new immunodeficient TBI rat model and transplanted human NSCs to the rat model, the recovery of cognitive function after brain injury was observed for a long time (greater than or equal to 2 months), and 9 to 25% of NSCs transplanted into the TBI model survived for at least 5 months, and differentiated into mature neurons, astrocytes, and oligodendrocytes; this study suggests that transplanted human NSCs may be an effective, long-term treatment for neurological recovery after brain injury. In addition, transfection of genes that promote growth into NSCs could enhance the proliferation, differentiation and other functions of NSCs, and then transplantation of these NSCs into the TBI model is an effective method for the treatment of TBI. In a study by Philips et al. (2001), injection of NSCs transfected with a virus carrying a nerve growth factor gene into the TBI model significantly increased pyramidal cell survival in the hippocampus, and enhanced the ability of cognitive, learning, and motor function. At present, NSCs are transplanted into animal models mainly through stereotactic injection and lateral ventricle injection, and NSCs injected through the lateral ventricle have higher survival rates *in vivo* (Wallenquist et al., 2009). Despite the NSCs transplantation method, the time of transplantation is also a key factor affecting the efficacy of NSCs treatment of TBI. The effect of injecting NSCs 2 days and 1 week after TBI is significantly better than that after two weeks, and injecting NSCs only 1 month after TBI has no significant effect for the recovery of motor and cognitive function (Zhang et al., 2005). At present, the application of NSCs to clinical trials is mainly limited by the difficulty in large-scale cultivation and production of NSCs. Recently, there is a clinical study on the treatment of chronic cervical spinal cord injury using human NSCs (Ghobrial et al., 2017). Although the study found that neurological function was generally restored after treatment with NSCs, it was necessary to increase the sample and add control group to further clarify its efficacy.

### Multipotent Adult Progenitor Cells

Multipotent adult progenitor cells were first reported in 2002, and received attention because of their characteristics of differentiation into mesenchymal cells, visceral mesoderm, neuroectoderm and endoderm (Reis et al., 2017). Their differentiation potential is not sustained (Reis et al., 2017). MAPCs are a distinct group of cells that differ from MSCs because of the low expression of MHC class I surface proteins and their ability to differentiate into endothelial cells (Roobrouck et al., 2011). To date, only one research team has used SD rats (Walker et al., 2010; Bedi et al., 2013a) and mice (Walker et al., 2012) to study human MAPCs for TBI. They injected 106 MAPCs into the TBI animals at 2 and 24 h after TBI. It was found that MAPCs could improve their spatial learning, information retention, memory retrieval and dyskinesia after 120 days of brain injury, and could maintain the integrity of the BBB in the acute phase of TBI (Bedi et al., 2013b). The main mechanism may be counteracting the inflammatory response caused by injury by up-regulating the expression of anti-inflammatory response factors (Bedi et al., 2013a).

### Induced Pluripotent Stem Cells

Induced pluripotent stem cells (iPSCs) were first reported in 2006. Two Japanese scientists used viral vectors to transfer the transcription factors Oct3/4, Sox2, c-Myc and Klf4 into differentiated somatic cells and reprogram the cells into a class of cells resembling embryonic stem cells (Takahashi and Yamanaka, 2006). Such cells can be extracted from patients and then reprogrammed *in vitro* to generate iPSCs, which are then returned to the patient, thereby avoiding ethical problems and immune rejection. They have the ability to self-renew and differentiate into various types of cells, so have promising clinical application prospects. Recently, Cary et al. (2015) obtained iPSCs by taking a dura mater from a patient with severe cognitive impairment after TBI, and then extracted fibroblasts from the tissue, finally iPSCs can be obtained by transfection of Sendai virus carrying non-conformity. For patients with brain trauma requiring surgery, taking the dura mater for culture is a good way to obtain iPSCs. Kobayashi et al. (2012) used the spinal cord injury marmoset model, the authors found that transplanted human iPSCs could survive in animals and differentiate into three neural cell lines, which promoted axonal regeneration and prevented brain tissue damage. Gao et al. (2016) used retrovirus to reprogram four transcription factors Oct4, Sox2, Klf4 and c-Myc and reactive glial cells into iPSCs, and found that such iPSCs could differentiate into a large number of NSCs and could be further differentiated into neurons and glial cells, repairing TBI damaged brain tissue. Lyu et al. (2017) used the TBI model and found that iPSCs-derived A2B5+ cells could effectively improve neurological dysfunction after transplantation into the brain injury zone. The mechanism is mainly related to the change of lncRNA and mRNA expression. Similarly, Wei et al. (2016) and Dunkerson et al. (2014) used animal TBI models and found that iPSCs transplantation could significantly improve cognitive and motor function after TBI. In addition to promoting the recovery of neurological function after trauma, iPSCs is also an important choice for the treatment of Huntington’s disease. An et al. (2012) used the human Huntington’s disease cell model to study and extracted fibroblast reprogramming from Huntington’s disease patients to finally obtain iPSCs, and found that iPSCs could change the phenotype of Huntington’s disease cell model, and could further differentiate into striatum neurons. Although iPSCs have many advantages, there are still many shortcomings (Dekmak et al., 2018). First, since this cell is reprogrammed by infection with a virus, it has certain tumorigenicity, and its efficiency from reprogramming by somatic cells is low. Furthermore, as such cells generated by reprogramming have an unknown genetic and epigenetic background, the safety issues need to be carefully evaluated before using iPSCs in the clinic.

### Endothelial Progenitor Cells

Endothelial progenitor cells are precursor cells of vascular endothelial cells, mainly in the bone marrow. They are progenitor cells with migratory properties that can be further differentiated into vascular endothelial cells. They can participate in embryonic angiogenesis and postnatal angiogenesis. Under the stimulation of physiological or pathological factors, EPCs can be mobilized from the bone marrow into peripheral blood by certain chemokines and adhesion molecules, spontaneously recruited to the site of endothelial injury, and participate in endothelial repair (Malinovskaya et al., 2016), especially in the brain after trauma (Guo et al., 2009). Changes in the number of peripheral blood EPCs can be used as markers of whether the BBB is destroyed (Huang S. H. et al., 2013). In addition, EPCs were significantly elevated within 24 h after TBI, whereas patients with relatively low levels of EPCs in peripheral blood could suggest a poor prognosis (Lin et al., 2017). BBB is mainly composed of microvascular endothelial cells. Boyer-Di Ponio et al. (2014) found that certain conditions can induce EPCs to differentiate into BBB. Endothelial colony-forming cells (ECFCs), also known as “late endothelial progenitor cells” or “endothelial outward-growth cells,” are a subtype of EPCs, first reported by Lin et al. (2000). In the brain injury model, ECFCs injected into the umbilical cord blood through the ventricle were found to be able to home to the brain injury area to participate in the repair of BBB and enhance neovascularization (Huang X. T. et al., 2013). Activation of the Notch pathway enhances the migration and lumen formation of EPCs, and activation of the Notch pathway of EPCs in the TBI animal model promotes the repair of damaged blood vessels and brain tissue (Ran et al., 2015). In order to track the distribution of EPCs in injured brain tissue and to clarify whether EPCs actually migrated to damaged brain tissue for repair, some scholars used GFP and BrdU double-labeled EPCs, find that EPCs can home to damaged brain tissue after intravenous injection, and promote hippocampal neurogenesis and angiogenesis, ultimately improving neurological function after TBI (Guo X. B. et al., 2017). Xue et al. (2010) studied EPCs isolated from adipose tissue and found that EPCs can accumulate in damaged brain tissue and participate in the capillary formation, reduce astrocyte proliferation and inflammation. Similarly, Chen et al. (2013) injected SPIO-labeled EPCs into the TBI model 6 and 12 h after TBI, respectively, and found that after 1 week, the cerebral blood perfusion recovered and the microvessels increased significantly in the damaged brain tissue area. Because the number of EPCs in peripheral blood is extremely small, transplantation of EPCs *in vitro* may be an effective method to promote neurogenesis and neurological recovery in patients with TBI. In addition, exogenous drugs enhance the migration ability and lumen formation of peripheral blood EPCs, can promote the recovery of neurological function after TBI, such as erythropoietin (Wang et al., 2015) and progesterone (Yu et al., 2016). The reduction of white matter after TBI is an important indicator of survival and prognosis of patients (Carmeliet and Tessier-Lavigne, 2005). Blood vessels provide structural support for the growth and development of axons, as well as transporting oxygen and removing metabolic waste. In view of the important role of vascular cells in axonal development and homeostasis, some scholars suggested that EPCs played a role in mediating local angiogenesis in the brain, and used TBI animal models to confirm that EPCs could maintain the integrity of white matter after TBI and reduce capillary damage (Park et al., 2014). At present, although a large number of basic experiments have confirmed that EPCs transplantation therapy can significantly improve neurological function after TBI, its efficacy and safety remain to be further studied.

## Conclusion and Prospects

Although there is a large volume of basic research into TBI, especially on the complexity of pathophysiology and the application of stem cell therapy, there are still many problems that need to be solved, to determine the best method for brain function recovery. Although there have been clinical studies on stem cell treatment of TBI, and all have achieved good therapeutic results, the sample size is not large enough, and there is no control group. Therefore, all studies and interventions that may affect the efficacy of TBI treatment require multi-center long-term follow-up and randomized prospective trials, which will have a huge impact on our decision to develop appropriate treatment options for different TBI populations. Although a large number of basic studies have confirmed that stem cells have good effect in the craniocerebral injury, the safety of stem cells, the route of injection, the time of injection and the specific mechanism are all factors that affect the clinical application of stem cells., and are the important research point in the future study.

## Author Contributions

YZ, AS, WX, and HW reviewed the literature and drafted the manuscript. AS and YD finalized the manuscript and provided suggestions to improve it. YZ and AS critically revised the texts and figures. All authors participated in the designing of the concept of the manuscript, and read and approved the final manuscript.

## Conflict of Interest Statement

The authors declare that the research was conducted in the absence of any commercial or financial relationships that could be construed as a potential conflict of interest.
